# Intergenerational income distribution before and after the great recession: winners and losers

**DOI:** 10.1007/s40622-022-00325-w

**Published:** 2022-09-28

**Authors:** Filip Chybalski

**Affiliations:** Institute of Management, Lodz University of Technology, Wólczańska 221, 93-005 Lodz, Poland

**Keywords:** Income distribution, Inequality, Public policy, Great recession, Intergenerational relations, Ageing, D31, D63, E24, I32, J18

## Abstract

Ageing process may affect intergenerational relations in different ways, including income distribution. Simultaneously, the global economy has undergone various crises. An important question is whether these crises impact the nexus between ageing and intergenerational income distribution. Finding an answer to this question is difficult in the literature; although the crisis-income inequality nexus is quite often investigated, this is not in the intergenerational context. This paper attempts to solve such puzzles using data covering 13 OECD countries in the period 1995–2018. The findings show that the relationship between the age structure of the population and intergenerational income distribution before and after the Great Recession of 2007–2009 was quite different. Actually, the Great Recession seems to have triggered this nexus in such a way that the elderly won the intergenerational income game in the aftermath of the crisis; however, the working-age and younger cohorts took a rematch later. The results obtained may support the political decision-making how to cope with economic crises, including the present COVID-19 pandemic and its impact on the economy and society, so as to treat different generations fair.

**Supplementary Information:**

The online version contains supplementary material available at 10.1007/s40622-022-00325-w.

## Introduction

The topic of the nexus between financial crisis and changes to income distribution or income inequality is quite often addressed in the literature. Two main strands of investigation within this area can be observed. The first examines whether income inequality leads to credit booms and financial crises (for review: Kirschenmann et al. 2016; Kumhof et al. 2015; Perugini et al. 2015; Bordo and Meissner 2012). The second strand addresses a reverse relationship and embraces studies in which the impact of financial crises on the change in income distribution across population is studied (for review: Smeeding 2012; Callan et al. 2014; Grabka 2015; Pfeffer et al. 2013; Gokmen and Morin 2019; Wolff 2013). From the perspective of this paper, the latter literature vein is substantial; however, the studies within this area lead to some inconsistent conclusions in terms of the impact crises have on income inequality. In regard to the Great Recession 2007–2009 (the GR hereinafter), many studies address the mentioned nexus for US data, while the problem is tackled less often for the European countries, although the GDP declines affected the Old Continent as well. However, investigations which struggle to solve this puzzle usually disregard the problem of changing intergenerational relations in the era of an aging population. On the other end of the pole, there are many studies which tackle the problem of intergenerational inequalities in the demographic context without any special attention paid to the GR For review: Chauvel and Schröder 2014; Vanhuysse 2014; Tremmel and Vanhuysse 2019 and demonstrate that the changing age-structure of the population affects income distribution across generations. Since the mentioned studies on the nexus between financial crises (including GR) and income distribution as well as studies on the nexus between intergenerational inequalities and demographic environment (especially aging) have been conducted separately, this paper attempts to bridge them. The reason is that it remains unclear how the GR affected the income distribution across generations taking the demographic context into account. To solve this puzzle and answer the question whether the nexus between the age structure of the population and income distribution across generations changed in the time of the GR, a cross-sectional and time-series analysis of 13 European OECD countries in the period between 1995 and 2018 is developed.

The paper contributes to the literature in the following ways. First, although previous literature focuses on the impact economic crises have on income distribution measured across different income groups, this study attempts to capture this nexus, but from a new—intergenerational perspective referring to different age groups and taking demographic context into account. Second, the great majority of studies in the investigated field date from the beginning of the 2010s, having analysed the income trends directly after the GR. Such a short period, however, does not allow one to recognize the nature of the trends identified. From the perspective of today, it is important to resolve whether these tendencies are only short-term in character, or whether they have stronger foundations and are more stable over time. This study attempts to avoid this limitation as is it based on data covering a long period before and after the GR. For this reason, it sheds a new light on the intergenerational income distribution before and after the crisis. Third, the paper additionally tries to identify the main direct drivers of the changes to income distribution across age groups taking the changes in the labour market, pension system generosity and some policy measures applied into account. The study may also support public policy decisions-making as the results show that the GR changed intergenerational income distribution and this deteriorated more for the population aged under 65 than for generation aged 65 and over. This suggests that in case of future crises, a better cushion should be prepared for the working-age population and their children. This refers to the present time and to the economic consequences of COVID-19 pandemic as well.

The remainder of the paper is structured as follows. The next section presents the literature review where the contribution to the topic on the nexus between financial crises or economic recessions and income distribution are discussed. Then, the research procedure, data and the results of empirical study are presented broadly. The paper ends with the discussion of the findings and summary conclusions.

## Literature review

The aging population stimulates the global discussion on intergenerational fairness or justice perceived through the prism of many aspects of our lives. Income is one of the most important and frequently discussed among them. The vast body of literature demonstrates that the young or working generation is in a worse situation than the elderly in this respect. One of the reasons behind this may be gerontocracy caused by ageing (Montén & Thum, 2010) and manifested in the political power of older voters. As Magni-Berton and Panel (2021) point, ‘older voters, which participate more in politics, tend to prefer older politicians, because they (correctly) expect them to better defend their own interests’. Thus, gerontocracy as the results of ageing can lead to income distribution that is beneficial for their generation at the expense of the younger cohorts. However, the views on the real significance of gerontocracy and its impact on the socio-economic policy of contemporary democracies are not unified. To review, while Atella & Carbonari (2017) claim that “the damage caused by gerontocracy” as an effect of the aging population, is harmful for growth due to long-term delayed return on investment (in public education or productive government services), Vanhuysse (2015) argues that “demography is not destiny” and good policy can mitigate the impact the aging process has on intergenerational relations. Obviously, demographics are a challenge and impede the formation of fair intergenerational relations. However, the question is whether governments are able to implement policies that support a kind of intergenerational balance ensuring both adequate pensions and public services for the elderly on the one hand, and growth and good prospects for the working and young generations on the other hand. A separate vein of literature includes deliberations on the impact a model of welfare state has on generational policies across contemporary democracies (Chauvel & Schröder, 2014; Chłoń-Domińczak et al., 2019; Goerres & Vanhuysse, 2012), sometimes also taking the aspects of (de)familization or (de)genderization into account (Albertini et al., 2007; Daatland et al., 2012; Folbre & Wolf, 2013; Saraceno & Keck, 2010), or stressing a growing state support for families (Ferragina, 2019; Ferragina et al., 2013; Gauthier, 2002; Thévenon, 2011). Thus, the impact of demographics on the intergenerational distribution of incomes or wealth is very complex and can be moderated by both welfare state policy as well as intra-family behaviours.

Although demographics as well as policy design matter with regard to intergenerational relations, the question whether in the period of GR has something changed in this respect remains unanswered. The impact of the crisis on income distribution is not an unusual topic in economic literature and the great majority of studies in this area focuses on the inequality across population referring to different income groups (quantiles, deciles). They draw, however, a somewhat fuzzy picture of this nexus. In regard to Americans, Pfeffer et al. (2013) show that a decline in wealth were noted by all socioeconomic groups. Wealthier Americans lost more than poorer ones in absolute terms, whereas poorer ones lost more in relative terms. Similarly, Smeeding (2012) indicates a middle wealth class as one that recorded the highest loss of wealth in relative terms, mainly due to decreases in the housing market. As for the income and wealth inequality, both have increased over several decades and there is little chance that the financial crisis would change this trend (Pfeffer et al., 2013). However, Wolff (2013) demonstrates that although wealth inequality increased in the United States during or directly after the GR, a slight decrease in income inequality was recorded. Nevertheless, in regard to both income and wealth, a long-term trend of growth in the Gini coefficient seems to exist between 1983 and 2010.

As for cross-country studies or non-USA country case studies, to find a universal solution to the puzzle of the nexus between the financial crisis and income or wealth distribution also seems to be a challenge. Gokmen and Morin (2019) analyse 70 countries using data between 1973 and 2006 and find that there is not a general rule how income inequality changed in the aftermath of financial crises since the type of a crisis matters. They conclude that whereas in the case of advanced economies after stock market crises income inequality decreased, in the case of emerging countries this was not observed. An important finding is that stock market crises reduced wealth across households in top income quantiles, having alleviated inequality this way. Jenkins et al. (2012) study national accounts of selected OECD countries and show that despite GDP decline between 2007 and 2009, in the case of some countries, gross household disposable income not only did not decrease, but even increased. This was largely caused by the political decisions to support mainly low-income groups. Simultaneously, capital gains, which are an income source for mainly high-income households, decreased. These two factors contributed to the reduction of income inequality across different income groups. They finally conclude that the short-term impact of the crisis on income distribution was rather weak; however, they expect the long-term impacts to be greater and more diverse across countries, which may be the result of fiscal consolidation measures applied as a consequence of the GR.

The case study of Germany, which is undoubtedly an advanced economy, demonstrates that the crisis-inequality nexus was not observed. Moreover, empirical data give the impression that “the Great Recession temporarily froze the income structure”, however, “afterwards income mobility tries to make up leeway” (Grabka, 2015). Callan et al. (2014) investigate a direct as well as modified (by policy measures) nexus between crisis and income distribution in Ireland. In this case, although income inequality was stable from early 1990s until the crisis (2007), it tended to decrease between 2007 and 2009. Then the Gini coefficient increased in 2010, but shortly after, in 2011, decreased. Callan et al. additionally analyse the poverty measure (percentage of people below 60% of median income) across different age groups. The data between 2005 and 2011 show that the productive-age group recorded the greatest increase in the poverty between 2009 and 2011. In case of the elderly, poverty decreased between 2009 and 2010 and then increased between 2010 and 2011 attaining the previous level (from 2009). In the case of children, it remained stable between 2009 and 2011. The greatest level of poverty characterized the youngest group, whereas the lowest poverty was observed across the elderly. The changes of income inequality corresponded with some policy measures. Its decrease in 2009 was accompanied by an increase in welfare payments and an increase in taxes and levies in the same year. After 2009, income inequality increased, which corresponded with public expenditure retrenchment. Savage (2018) shows that what contributed to income decline among the poorest in case of some European countries (Greece, Spain, Italy and Estonia) was the mobility across the income distribution rather than income losses for individuals who came into the GR in the bottom decile.

The literature overview indicates that quite much is known on how economic or financial crises affect income distribution or inequality across different income groups. Meanwhile, our knowledge on how crises (the GR particularly) affect the income distribution across generations (or age groups), is very poor. In the further part of this paper, I shall focus on this issue distinguishing simplistically between two generations perceived as different age groups (following the chronological-temporal approach to define generations by Tremmel 2014)^1^: pensioners (population aged 65 years and over) and the remaining population (population aged 0–64 years). Due to data constraints for a long time period, it was impossible to extract three generations in the empirical study (pensioners, working-age and youth). Additionally, I account for the demographic trends which in case of possible gerontocracy could affect the intergenerational income distribution in the analysed period. This way, the changes of income distribution across two age groups mentioned are analysed with reference to two-dimensional landscape – the Great Recession and population ageing.

## Empirical study

### Data and methods

In the empirical study, I try to examine whether the relationship between the age structure and income distribution changed around the GR period, and if so, what the possible factors behind it were. The indicators used in the analysis are defined in Table 1. The dependent variable is the relative median income ratio (*RMI*) which is the quotient between the median equivalised disposable income of people aged over 65 (*MI65* +) and the median equivalised disposable income of those aged under 65 (*MI65-*). Thus, the concept of this measure is based on the division of the population into two separate age groups: aged under 65 years and aged 65 years and over. That is why in stage 3 (see below), I try to use the explanatory variables for these two age groups separately. As explained by Eurostat, equivalised disposable income is the total income after taxation and other deductions. Thus, it includes all the monetary income received by a household from any source, not only from work or the pension system. Therefore, the relative median income ratio is a more comprehensive measure in comparison to the aggregate replacement ratio, as the latter accounts only for pension benefits. Moreover, *ARR* is expressed in gross value, not in net value, as *RMI* is. The equivalised disposable income accounts for household size to ensure that it has better comparison properties.^2^ Thus, the relative median income ratio can be a good measure to compare the net disposable income of people aged 65 and over and people aged under 65 regardless of household size and structure. In this paper, I simplify, so that population aged 65 and over reflects the pensioners generation, whereas population under 65 reflects the generation of the working-age generation, youth and children. This simplification is forced by data constraints. Namely, *RMI* (conceptually referring to age groups under 65 and 65 and over) is an income measure that is comparable across countries, aggregated to macro level, and covers over ten countries and period of over 20 years. Although equivalised household income can serve for comparisons of individuals or households in terms of their welfare, it has some limitations. Especially, they relate to the use of longitudinal data, as household composition can evolve over time. This results in the change of weighting factors which vary for household members representing different age groups (United Nations, 2011). Nevertheless, disposable equivalised income is said to be a good measure of individual economic well-being (Raitano, 2016).

**Table 1:** Characteristics of control variables. *Source:* Own elaboration on the basis of OECD and Eurostat information

Indicator	Description	Source of data and definition
Variables dedicated for each gender separately		
RMI	The ratio of the median equivalised disposable income (*MI*) of people aged above 65 to the median equivalised disposable income of those aged below 65	Eurostat
65 + /65-	The ratio between population aged 65 and over, and population aged under 65	OECD
MI (65-, 65 +)	The total median income of a household, after tax and other deductions, that is available for spending or saving, divided by the number of household members converted into equalised adults; household members are equalised or made equivalent by weighting each according to their age, using the so-called modified OECD equivalence scale	Eurostat
ARR	The ratio of the median individual gross pensions of 65–74 age category relative to median individual gross earnings of 50–59 age category, excluding other social benefits	Eurostat
ARP (65-, 65 +)	The share of persons with an equivalised disposable income below the risk-of-poverty threshold, which is set at 60% of the national median equivalised disposable income (after social transfers)	Eurostat
SMD (65-, 65 +)	The percentage of the population that cannot afford at least four of the following nine items: to pay their rent, mortgage or utility bills; to keep their home adequately warm; to face unexpected expenses; to eat meat or proteins regularly; to go on holiday; a television set; a washing machine; a car; a telephone	Eurostat
UNEMP (15–24, 20–64, 55–74)	The number of unemployed persons as a percentage of the labour force based on International Labour Office (ILO) definition. The labour force comprises total number of people who are employed or unemployed. Unemployed persons are those in a given age group (15–24, 20–64 or 55–74) who:—are without work during the reference week;—are available to start work within the next two weeks;—and have been actively seeking work in the past four weeks or had already found a job to start within the next three months	Eurostat
AER	The average age of all persons withdrawing from the labour force in a given period^1^	OECD
GDP_PC	Gross domestic product per capita in thousands of PPS (measured in current prices)	Eurostat

^1^A detailed description of the calculating methodology is available at http://www.oecd.org/els/emp/39371923.pdf (retrieved on January 8, 2019)

In the analysis, data from Eurostat (EU-SILC, LFS) and OECD are used. The dataset covers Austria (AT), Belgium (BE), Finland (FI), France (FR), Germany (DE), Greece (GR), Ireland (IE), Italy (IT), Luxembourg (LU), Netherlands (NL), Portugal (PT), Spain (ES) and United Kingdom (UK) in the period 1995–2018. The data set (both in terms of countries selected as well as period covered) is determined by data availability. Nevertheless, some data gaps exist in this set. To cope with gaps referring to *RMI*, the year 2002 is excluded from the analysis. Furthermore, the following procedures in regard to *RMI* data gaps are employed:

- Finland: the gap for 1995 was replaced by data from 1996,
- France, Italy, Portugal, Spain and United Kingdom: the gap for 2003 is estimated as a simple mean of *RMI* from 2001 to 2004,
- Germany, the Netherlands: the gaps for 2003 and 2004 are estimated as a simple mean of *RMI* from 2001 to 2005.

In the case of data used to develop Figs. 4–8, data gaps were ignored when calculating the mean values. Such data gaps were recorded in 2003 and/or 2004 in case of *MI65-*, *MI65* + , *ARP65-*, *ARP65* + , *SMD65-*, *SMD65* + . For the same reason, France was omitted in the calculation of mean values of *Unemp* (15–24, 20–64, 55–74) between 1995 and 2002. Since the primary attention is paid to the period around the GR, these gaps do not affect the most important results.

The procedure employed in the empirical analysis consists of the following stages:

1. Regression models for paned data are estimated where the relative median income ratio (*RMI*) is a dependent variable and age structure (*65* + */65-*) consistent with the concept of *RMI* is the main predictor, i.e. in both indicators the whole population is divided into two subsets – people aged under 65 and people aged 65 and over. The inclusion of the *65* + */65-* indicator is motivated by the goal of this paper, which is to examine whether the intergenerational income distribution changed at the time of GR taking the demographic context into account. A possible nexus between *RMI* and *65* + */65-* has theoretical grounds in the causal relationship between ageing and gerontocracy (as discussed in Sect. 2). This nexus can deliver some information whether, before or after the GR, the growing political power of the elderly (reflected in their proportion of the whole population) resulted in the relative improvement of their economic situation (reflected in *RMI*). If a positive relationship between these two variables is identified, this would be a manifestation of gerontocracy in economic terms. In the estimated models, I control for average effective age of retirement (*AER*) and GDP per capita (*GDP_pc*). The first variable may affect the income of retirees especially in defined contribution (DC) pension schemes, but also in earnings-related defined benefit (DB) pension schemes. GDP per capita, as a measure of the general welfare of the population, may affect incomes of both pensioners (proxied by population aged 65 and over) and people at working age, youth and children (proxied by population aged under 65).
  The panel regression models are commonly used to investigate socio-economic phenomena at a cross-country macro level. The method employed is based on panel regression models estimated for cross-country data. Such an approach is adopted in the literature to study various socioeconomic relationships (Fuinhas et al., 2015; Hong & Knapp, 2014; Schmidt-Hebbel & Serven, 1997; Tas et al., 2013). Two types of panel regression models are estimated: with fixed (FE) and with random (RE) individual effects. The FE estimator is used to account for some factors that are difficult to measure and to include in the model explicitly. The RE estimator is used mainly to increase the estimator efficiency. The FE as well as RE estimators reduce the omitted-variable bias caused by the aware or unaware omission of some controls, which is possible due to the inclusion of individual effects. This is an important strength of such models in comparison to cross-sectional or time-series regression as they allow for the reduction of control variables and, as a consequence, maintaining a greater number of degrees of freedom. On the one hand, intuition suggests that with regard to the phenomena under analysis, the FE estimator should be employed at first, since some implicit economic, social or political factors may determine the intergenerational distribution of incomes. Moreover, as Baltagi (2013) indicates, FE estimator is appropriate for a specific set of objects investigated, e.g. firms or countries, whereas the RE estimator should be used for the samples randomly selected from a large population (which is not case in this study). However, due to methodological caution, both FE and RE estimators are used as well as the Wald test, the Breusch-Pagan test, and the Hausman test are employed (Baltagi, 2013; Wooldridge, 2010) to compare the estimates obtained.

2. All the countries studied are graphically mapped in terms of the change in age structure (*65* + */65-*) and intergenerational income distribution (*RMI*). Two scatter plots are developed. The first one shows the movement of countries in two dimensional space (*65* + */65-* and *RMI* dimensions) between 1995 and 2008, the second one between 2009 and 2018. In this way, countries are classified to different groups in terms of the changes investigated.

3. The average values of the selected indicators are calculated for each cross-section (including all the countries studied). In this way, their time series are obtained that allow identification of possible time coincidence of the changes experienced by *RMI* and some other variables, which can explain why the observed trends around the GR period exist. The variables used in this stage are as follows: median equivalised disposable income for age groups 65- and 65 + (*MI65-*, *MI65* +), average effective age of retirement (*AER*), aggregate replacement ratio (*ARR*), unemployment rate in age groups 15–24, 20–64 and 55–74 years (*Unemp15-24*, *Unemp20-64* and *Unemp55-74*), at-risk-of-poverty rate for age groups 65- and 65 + (*ARP65-*, *ARP65* +) and severe material deprivation rate for age groups 65- and 65 + (*SMD65-*, *SMD65* +). The analysis is conducted for the period 1995–2018 (with an exception of *ARR* and *SMD*, in case of which the period is limited to 2003–2018 due to data constraint)

4. Time series for the proportion of people aged 65 and over in the population (*Elderly*), average effective age of retirement (*AER*), GDP per capita (*GDP_pc*) and relative median income ratio (*RMI*) across countries studied between 1995 and 2018 are analysed with the use of graphs. This allows one to find some cross-country variation in terms of the trends in ageing and intergenerational income distribution while controlling for GDP per capita and average effective age of retirement.

For time series analysis, the Chow test for structural break is employed in stages 4 and 5 (Chow, 1960). For a period assumed to be known a priori (2008 in our case), the data set is divided into two subgroups (1995–2008 and 2009–2018). Three separate models are estimated: the first one for the whole period of *n* observations, and two other for the subperiods of *n*_1_ and *n*_2_ observations (*n* = *n*_1_ + *n*_2_). The null hypothesis that the parameters of the two models estimated for subperiods are equal is tested using the following statistic:

$$F=\frac{\left[\mathrm{RSS}-{(\mathrm{RSS}}_{1}+{\mathrm{RSS}}_{2})\right]/(k)}{{(\mathrm{RSS}}_{1}+{\mathrm{RSS}}_{2})/\left({n}_{1}+{n}_{2}-2k\right)}$$

where RSS, RSS_1_ and RSS_2_ denote the sum of squared residuals for the models estimated for the whole period and for two subperiods, respectively, and *k* denotes the number of parameters estimated. This *F* statistic follows the *F*-distribution with *k* and *n*_*1*_ + *n*_*2*_-2* k* degrees of freedom.

### Results

**Fig. 1:**
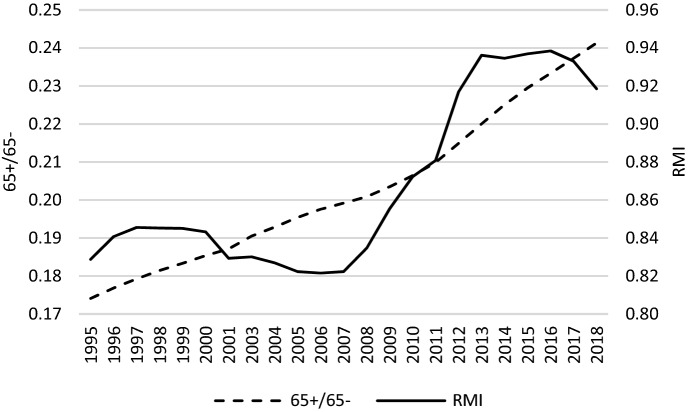
*RMI* and *65* + */65-* across countries studied – a glance on average values. *Source:* own computations on the basis of Eurostat data

Figure 1 shows that although in average terms an ageing process was observed across the countries studied over the whole period (1995–2018), the ratio between income of elderly (65 +) and younger cohorts (65-) started to grow from the GR, not before. This suggests that the elderly generation then improved its economic situation as compared to the generation of those aged 0–64 years. The Chow test for a time series of a mean value of *RMI* confirms a structural break in 2008 (for the results, see Table A1 in the Online Appendix).

The change in the relationship between 65 + /65- and *RMI* is an argument to analyse two periods separately: 1995–2008 and 2009–2018. To simplify, the former represents the period before the GR and the latter the period after the GR. The regression models estimated are presented in Table 2. The interpretation of parameters of both fixed and random effects models are similar as there are only some minor differences between them. Nevertheless, the results of Hausman test are consistent with methodological premises formulated on the basis of data set used and indicate the FE estimator as a better one for two periods studied at *p*-value < 0.10. The most telling point is that a positive nexus between the ratio 65 + /65- and relative median income ratio (*RMI*) is observed after the GR and not before. Moreover, the link holds even when controlling for average effective age of retirement (*AER*) that affects pension benefits positively (the later people retire, the higher the pension benefits they are paid; this is the case in DC schemes, but also in the majority of DB earnings-related schemes). Thus, the regression analysis suggests that something changed during/after the GR in terms of intergenerational income distribution. Namely, the ageing process started to correspond to the income distribution between the older and younger cohorts. After the GR, there was a time coincidence between the growing political power of the elderly (as voters) and increase in their incomes as compared to incomes of those aged 0–64 (changes of *RMI* reflects relative changes of incomes, not absolute ones).

**Table 2:** Models for RMI: before and after the crisis. *Source:* Own computations on the basis of OECD and Eurostat data

Models/Predictors	1995–2008				2009–2018			
	FE		RE		FE		RE	
Const	1.317	***	1.497	***	− 0.131		0.318	
**65 + /65-**	** − 0.171**		** − 0.122**		**1.450**	***	**1.202**	***
AER	− 0.007	*	− 0.010	***	0.013	**	0.005	
GDP_pc	− 0.001		0,000		− 0.002	*	0.000	
Test statistics								
Wald F^a^	41.752	***			44.888	***		
Breusch-Pagan chi^2			463.584	***			334.814	***
Hausman chi^2			6.605	*			12.163	***

*p*-value:* < 0.1, ** < 0.05, *** < 0.01

^a^for the models for 1995–2008: F(12, 153); for the model for 2009–2018: F(12, 114)

A bold values refer to the tested predictor which distinguised this variable (65 + /65-) from the other (control variables)

**Fig. 2:**
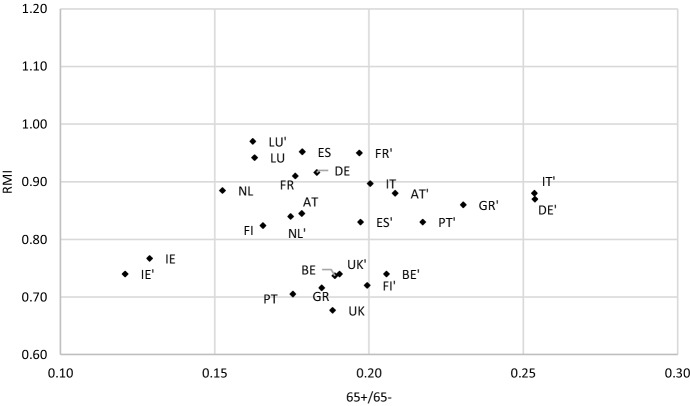
Countries studied in terms of *65* + */65-*ratio and *RMI* in 1995 and 2008* (*country name (e.g. IE) – 1995, country name’ (e.g. IE’) – 2008). *Source:* own computations on the basis of OECD and Eurostat data

**Fig. 3:**
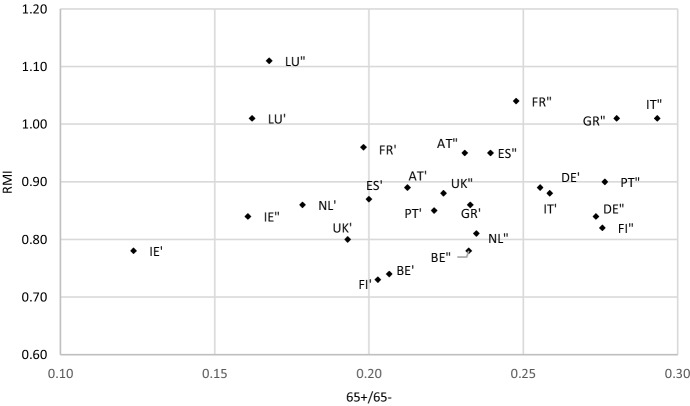
Countries studied in terms of *65* + */65-*ratio and *RMI* in 2009 and 2018* (*country name’ (e.g. IE’) – 2009, country name” (e.g. IE”) – 2018) *Source:* own computations on the basis of OECD and Eurostat data

Figures 2 and 3 present how the countries studied moved in the demographics-income distribution space. Figure 2 shows the change between 1995 and 2008, whereas Fig. 3 between 2009 and 2018. We can observe both demographic changes reflected by the *65* + */65-* ratio (horizontal axis), as well as income change reflected by the relative median income ratio (*RMI*). The two figures present a somewhat different picture. The movement of countries on the first is more chaotic than on the other, where the cloud of points relocates from lower to greater values of both indicators. Countries under analysis can be mapped to one of the following sets in terms of the change in the welfare of the elderly (for each period separately): elderly welfare retrenchment (EWR) which means a decrease in *RMI*, elderly welfare freezing (EWF) reflecting a stable value of *RMI*, or elderly welfare improvement (EWI) which corresponds with an increase in *RMI*. This change can be accompanied by different demographic conditions reflected by the *65* + */65-* ratio: increasing (↑65 + /65-), stable (s65 + /65-) or decreasing (↓65 + /65-). The result of this mapping developed on the basis of Figs. 2 and 3 is demonstrated in Table 3.

**Table 3:** Countries mapped in terms of elderly welfare change and demographic conditions change. *Source:* own elaboration

Country	Period 1995–2008	Period 2009–2018
Austria	EWI/↑65 + /65-	EWI/↑65 + /65-
Belgium	EWF/↑65 + /65-	EWI/↑65 + /65-
Finland	EWR/↑65 + /65-	EWI/↑65 + /65-
France	EWI/↑65 + /65	EWI/↑65 + /65
Germany	EWR/↑65 + /65	EWR/↑65 + /65
Greece	EWI/↑65 + /65	EWI/↑65 + /65
Ireland	EWR/↓65 + /65	EWI/↑65 + /65
Italy	EWF/↑65 + /65	EWI/↑65 + /65
Luxembourg	EWI/s65 + /65	EWI/s65 + /65
Netherlands	EWR/↑65 + /65	EWR/↑65 + /65
Portugal	EWI/↑65 + /65	EWI/↑65 + /65
Spain	EWR/↑65 + /65	EWI/↑65 + /65
United Kingdom	EWI/s65 + /65	EWI/↑65 + /65

Table 3 shows that between 1995 and 2008, thus until the middle of the GR, 7 out of 13 countries tried to freeze or even retrench the welfare of the elderly as compared to the rest of the population. Belgium and Italy kept the intergenerational distribution of income stable, and Finland, Germany, Ireland, the Netherlands and Spain even reduced income of the elderly as compared to people aged 0–64. Between 2009 and 2018, Germany and the Netherlands were the only countries which managed to keep the retrenchment of elderly income. In Luxembourg and the United Kingdom, before the GR the income of the elderly increased as compared to the rest of population, although the demographics were quite stable and the *65* + */65-* ratio did not change significantly. This suggests that the improvement of the welfare of the elderly was not accompanied by the growing political power of the voters aged 65 + as their population did not increase between 1995 and 2008 in these countries (see also Table A1 in the Online Appendix). After the GR, Luxembourg was the only country which managed to keep demographics stable, having continued to make the elderly more well off compared to the working population, youth and children. In Ireland, a trend reverse to ageing corresponded with the retrenchment of elderly’s welfare before the GR. Afterwards, Ireland followed the common trend to improve the relative income of the elderly while the population was ageing. However, the demographic starting point of this country was quite different to that of other countries. The age structure reflected in *65* + */65-* ratio was in Ireland much better, both before and after the GR.

To summarize the cross-sectional study, the main conclusion from the regression analysis is that the GR revealed the relationship between demographics and income distribution across the elderly and the rest of population. Namely, such a relationship was not observed in the period 1995–2008 and started to be the case afterwards, between 2009 and 2018. The analysis of Figs. 2 and 3, where countries are mapped in two-dimensional space in terms of demographic and intergenerational income distribution change, confirms this result. Before the GR, the countries studied presented various pictures of this nexus, while afterwards nearly almost all the countries (11 out of 13) experienced improvement of the welfare of the elderly under deteriorating demographics (with Luxemburg as the one exception where demographics have remained stable). The relative median income ratio before the GR generally assumed values between almost 0.7 and almost 1.0. After 2009, this interval moved to over 0.7 – over 1.0 (in Luxembourg even over 1.1). This means that a tendency to equalize the equivalized disposable income across age groups 65- and 65 + was observed. Thus, in countries such as France, Luxembourg, Greece and Italy, where the *RMI* was greater than 1.0 in 2018, income of the elderly was higher than income of the remaining younger cohorts.

**Fig. 4:**
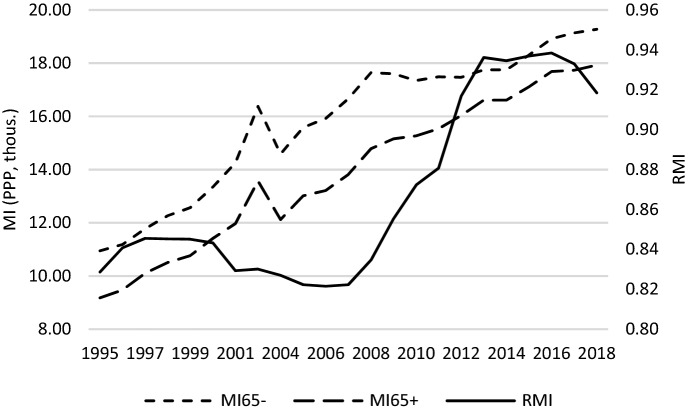
*MI* and *RMI* across countries studied – a glance at average values. *Source:* own computations on the basis of Eurostat data

**Fig. 5:**
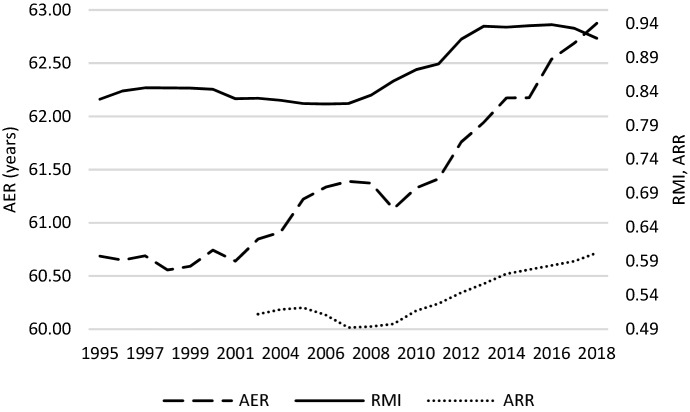
*AER*, *ARR* and *RMI* across countries studied – a glance at average values. *Source:* own computations on the basis of Eurostat data

**Fig. 6:**
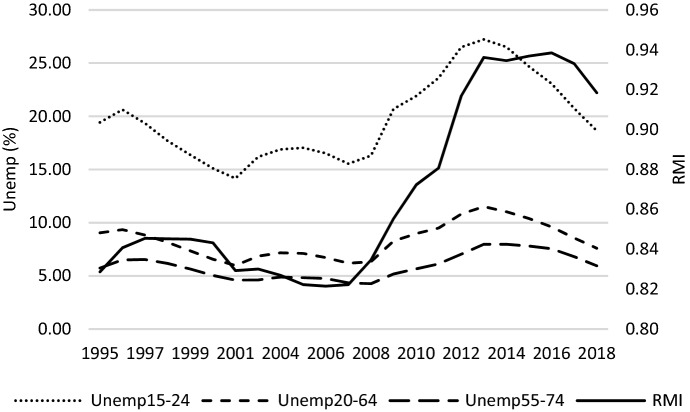
*Unemp* and *RMI* across countries studied – a glance at average values. *Source:* own computations on the basis of Eurostat data

In search of the reason behind these changes in relative median income ratio, Fig. 4 may be helpful. It shows that over the entire studied period there was a stable increasing trend of median equivalised disposable income in the two age groups under study (the period 2003–2004 should rather be ignored due to data gaps, as mentioned before). A slight decrease was observed in case of the population aged 65- between 2009 and 2010. Nevertheless, what shaped changes in *RMI* was generally the somewhat different dynamics of positive trends of median equivalised disposable income across the generations studied. What is observed in this regard is a damping increase in income of people aged 65- as compared to the elderly between 2009 and 2015. Income of the elderly expressed in purchasing power parity (PPP) was increasing a bit faster. Figure 5 shows that the reason behind this increase could be a growing aggregate replacement ratio (*ARR*), which means an increase in the gross pension benefits as compared to gross earnings. Although it decreased slightly between 2005 and 2008, it then started to grow. Moreover, in the period after the GR, this increase was accompanied by an increase in the average effective age of retirement (*AER*), which has an intuitive explanation—the later people retire, the higher the pension benefits they are paid. An increase in the average effective age of retirement was driven by changes in pensionable age. Many OECD countries decided to raise it in 2000s and 2010s. An additional explanation of the changes in *RMI* can be also delivered by Fig. 6 where unemployment rates are presented. We can observe that after the GR, the slightest increase in unemployment was observed in case of the age group 55–74; a bit stronger, however quite similar, in case of age group 20–64. Those who experienced negative consequences of the crisis the most were the youngest, in whose case the unemployment rate increased from about 15% to 25% in average terms (across the studied countries).

**Fig. 7:**
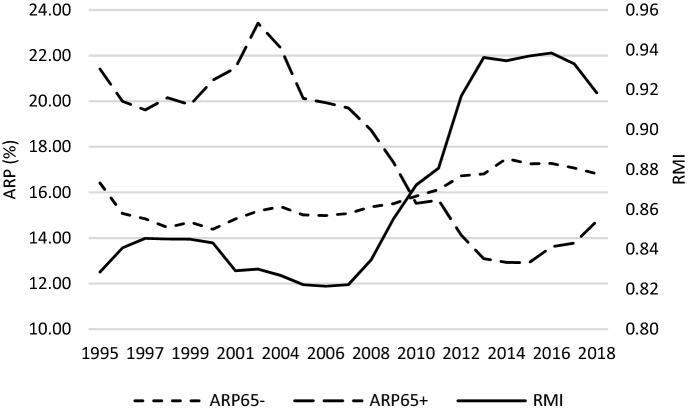
*ARP* and *RMI* across countries studied – a glance at average values. *Source:* own computations on the basis of Eurostat data

**Fig. 8:**
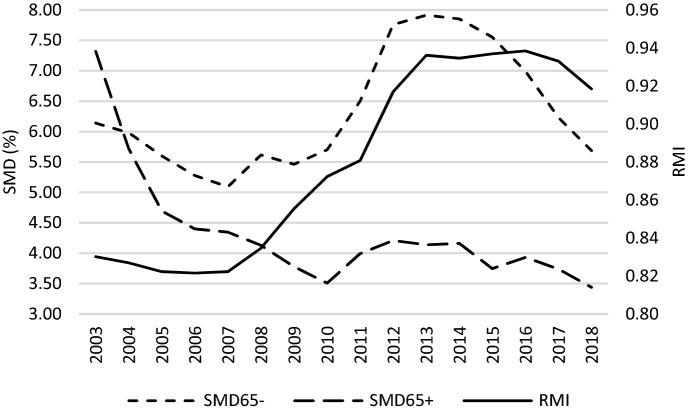
*SMD* and *RMI* across countries studied – a glance at average values. *Source:* own computations on the basis of Eurostat data

Figure 7 and 8 demonstrate how poverty changed, which can be treated as a socio-economic outcome of the GR caused by changes in income. Two measures of poverty are analysed. The at-risk-of-poverty rate (*ARP*) is a measure of relative poverty, as it assumes 60% of median equivalised disposable income as a cut-off point which makes this indicator vulnerable to the income inequality. Therefore, the severe material deprivation rate (*SMD*) as an absolute measure of poverty was used additionally. In the case of this indicator, poverty is measured as the inability to afford some needs (see definition in Table 1). With regard to *ARP*, during the crisis poverty among people aged 65- started to grow, while in case of people aged 65 + it continued a declining trend. Generally, the GR reversed the picture of poverty observed before it. Namely, before 2010 the elderly were poorer, while afterwards poverty was more frequently experienced by people aged 0–64. In terms of *SMD*, absolute poverty was lower among the elderly over the whole studied period (2003 and 2004 should be ignored due to data gaps). Both age groups experienced an increase in absolute poverty after the GR; however, in the case of the working-age population, youth and children, this increase was incomparably greater than in the case of the elderly. Then, between 2014 and 2018, absolute poverty entered into a decreasing trend in both age groups. What is common for the two figures analysed is the similarity of the poverty trends for the age group 65- to the trend of relative median income ratio. The data unambiguously demonstrate that an improvement of the welfare of the elderly (*RMI*) is accompanied by a deterioration of the economic situation of the age group 65- in terms of poverty (*ARP65-*, *SMD65-*). This is observed especially after the GR.

The cross-sectional study is complemented by a time series analysis for each country separately. The figures presenting the trend of the proportion of people aged 65 + (*Elderly*), average effective age of retirement (*AER*), GDP per capita (*GDP_pc*) and relative median income ratio (*RMI*) are included in Figure A1 (in the Online Appendix). They show that although some general tendencies or relationships can be captured at a cross-country level, some variations across countries are also observed. What is common for the great majority of countries studied is the change in intergenerational distribution (between age groups 65- and 65 +) favouring the generation of the elderly, which took place around the GR. In countries such as Austria, Belgium, Finland, France, Ireland, Italy Luxembourg, Spain or UK, there was a very clear retreat from the trend of freezing or even retrenchment of the welfare of the elderly measured by *RMI*, i.e. as compared to the population aged 65-. The dynamics of the changes in these countries was not similar, yet the directions were quite common. In the case of Greece or Portugal, a slow increase in *RMI* before the GR was continued after it. A separate group of countries consists of Germany and the Netherlands, where the retrenchment of the welfare of the elderly before the GR was also observed afterwards. Emphasis should be also placed on the fact that this was accompanied by a systematic increase in the average effective age of retirement. However, in all the countries under study stabilization or, in some cases, even a decrease in *RMI* was observed in the last 3–4 years. This means that there has not been a country where income of the elderly would have increased as compared to income of people aged 0–64 within the last years of the analysis.

The mentioned results of the trend analysis presented in Figure A1 (in the Online Appendix) are very consistent with the results of the Chow test for structural break for time series providing assumptions similar to those used previously for a mean value of *RMI* (for the results, see Table A1 in the Online Appendix). In case of the majority of countries (excl. Germany, Greece, the Netherlands and Portugal), the differences between models’ parameters are significant for *p*-value < 0.01. In the case of Germany and the Netherlands, the difference is statistically insignificant (*p*-value equal to 0.3245 and 0.9982, respectively). For Greece the difference is statistically significant (*p*-value < 0.02) which is consistent with the time series plot, which confirms a noticeable change within a positive *RMI* trend in the second subperiod. Portugal reports a statistically insignificant difference between parameters (*p*-value = 0.0762). This corroborates a stable positive *RMI* trend presented on a time series plot. Hence, the Chow test supports the hypothesis of a structural break in case of countries where a very clear retreat from freezing or even retrenchment of the welfare of the elderly as compared to the population aged 65- was observed. Three other countries were continuing a stable trend of *RMI* over the whole period. Greece is the only country that reports a significant change within a positive trend of *RMI*.

To summarize, both approaches – the one based on the cross-sectional data analysis as well as the one based on time-series analysis for each country separately – yield consistent results. The most important is that around the GR, income distribution across generations changed in favour of the elderly and discriminating against their children and grandchildren. In the last few years this process was stopped or, in some countries, even reversed. This all is accompanied by population ageing across the majority of countries investigated. Only Ireland, the United Kingdom and Spain between 1995 and 2008, and Luxembourg over the whole period studied were characterized by a stable proportion of people aged 65 and over.

## Discussion and conclusions

Generally (in average terms), the countries analysed experienced ageing over the whole period studied but the deterioration of the economic situation of population aged 0–64 as compared to population 65 and over was observed in the aftermath of the GR. This suggests that the crisis ‘triggered’ the nexus between ageing and income distribution for a few years (until 2013–2014). The change in the intergenerational income distribution is reflected in relative median income ratio as well as in poverty indicators, both relative and absolute. One of the obvious reasons behind this is that the working-age generation experienced a greater increase in the unemployment rate than the elderly. However, among the former, the most negative impact of the GR was observed among the youngest participants in the labour market. In their case, the increase in the unemployment rate was incomparably higher. The last five years of the analysis show a kind of reversal. Namely, first the relative median income ratio froze (between 2013 and 2016) and then started to decline. This means that directly after the crisis the elderly were favoured as compared to the younger cohorts, but then the income of the population aged 0–64 was growing more rapidly. The reason behind this is that a pension system ensures more stability and guarantee in terms of incomes in comparison to the labour market, especially in the relatively short run (e.g. of a few years).

Theoretically providing that optimal intergenerational income distribution is ensured when median equivalised disposable income for age groups 0–64 and 65 years and over is equal (i.e. relative median income ratio equals 1), the GR has reduced inequality in this regard. Something similar was observed e.g. by Gokmen and Morin (2019) or Jenkins et al. (2012) in developed countries; however, this was in reference to different income groups, not to age cross-sections. This paper also confirms observation by Jenkins et al. (2012) that in the aftermath of the GR, disposable income increased. Obviously, the assumption that *RMI* = 1 is optimal in terms of intergenerational income distribution is too simplistic, as households with different age structures have different needs. A good example are mortgage payments that burden a household’s budget more frequently in the working age population than in the pensioners generation. Pension savings accumulated by the working population (hence burdening the budget) and decumulating by the pensioners (hence expanding household’s budget) can serve as another example. This is limitation not only of this study. Median equivalised disposable income as calculated by Eurostat does not account for such a composition of needs and spending of households with different age structures. However, this limitation is more important when comparing income levels. In this paper, I rather put emphasis on income dynamics. Therefore, the conclusions are not biased by such a limitation significantly and clearly show who was the winner and who was the loser in this intergenerational game played directly after the GR. Later on, the picture changed, and the younger cohorts seemed to take the rematch.

Changes to the intergenerational income distribution that took place directly in the aftermath of the GR seem not to be stable over the long-term. Figure 4 demonstrates that median equivalised disposable income in the two age groups under study returned to the trend observed before the crisis. As a result, relative median income ratio also stabilized. This is inconsistent with prediction by Jenkins et al. (2012) that due to fiscal consolidation, the long-term impact of the GR on income distribution could be stronger than short-term ones. Although their work refers to income groups, whereas this study to age groups, fiscal consolidation after the GR was realized multidimensionally. In regard to intergenerational relations, it embraced inter alia pension reforms aiming to retrench spending on pension benefits, e.g. through an increase in the pensionable age. As Chybalski and Gumola (2021) demonstrate, although changes in the effective retirement age (perceived as an intergenerational borderline between pensioners and the working-age population) between 1971–2013 were deteriorating for the latter, the changes after 2000 were less unfavourable than those before. As it is argued, increasing retirement age supports economic growth and reduces the economic dependency of younger cohorts (Bauer & Eichenberger, 2016; Bernal & Vermeulen, 2014; Manoli & Weber, 2016; Peng & Mai, 2013; Staubli & Zweimüller, 2013). The fact that changes in the income distribution after the GR did not reverse the long-term trend in this respect seems to confirm that more and more countries have implemented policies that seek to cope with the aging process. This is an important change in comparison to what was done (or was not done) before 2000. This change is significantly delayed; however, better later than never.

**Fig. 9:**
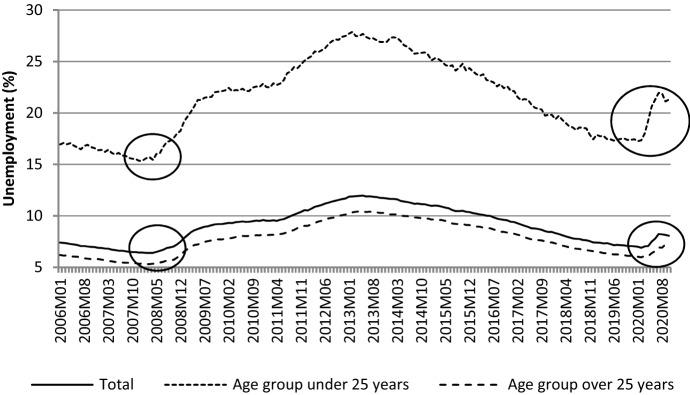
Unemployment (monthly data) around GR and around COVID-19 pandemic – a glance on average values. *Source:* own computations on the basis of Eurostat data

Last, but not least, the results obtained seem to have not only retrospective, but also prospective value, and therefore, can add to the present debate on what political decisions should be made to cope with the during-COVID-19 and post-COVID-19 crisis. As the empirical analysis demonstrates, an increase in unemployment varied across the age groups studied, which must have had consequences for different income dynamics in the population aged 0–64 years and in the population aged 65 years and over. Figure 9 shows that in the countries studied (in average terms) the similarity of changes in unemployment rate during the Great Recession (2007–2009) and in the first 6–7 months of crisis caused by COVID-19 pandemic is significant. The question is whether changes to income will also be similar. Future data should give the answer.

## Supplementary Information

Below is the link to the electronic supplementary material.

Supplementary file1 (PDF 687 kb)

## Footnotes

1 Generation in chronological-temporal terms means different age groups very often perceived as different social groups (young generation, working generation and generation of pensioners). This is one of few approaches to define generation proposed by Tremmel (2014).

2 More details are available at https://ec.europa.eu/eurostat/statistics-explained/index.php?title=Glossary:Equivalised_disposable_income (retrieved on November 27, 2020).
