# Supplementary material for: Intergenerational income distribution before and after the great recession: winners and losers
Source: Decision. 2022 Sep 28;49(3):311–27. doi: 10.1007/s40622-022-00325-w (PMC9518940; doi:10.1007/s40622-022-00325-w)
Supplement: Supplementary file 1 — Supplementary file1 (PDF 687 kb) [file 40622_2022_325_MOESM1_ESM.pdf]

Appendix (online supplementary material)

Figure A1. The proportion of people aged 65+ in the population (*Elderly*), average effective age of retirement (*AER*), GDP per capita (*GDP_pc*) and relative median income ratio (*RMI*) across countries studied between 1995 and 2018.

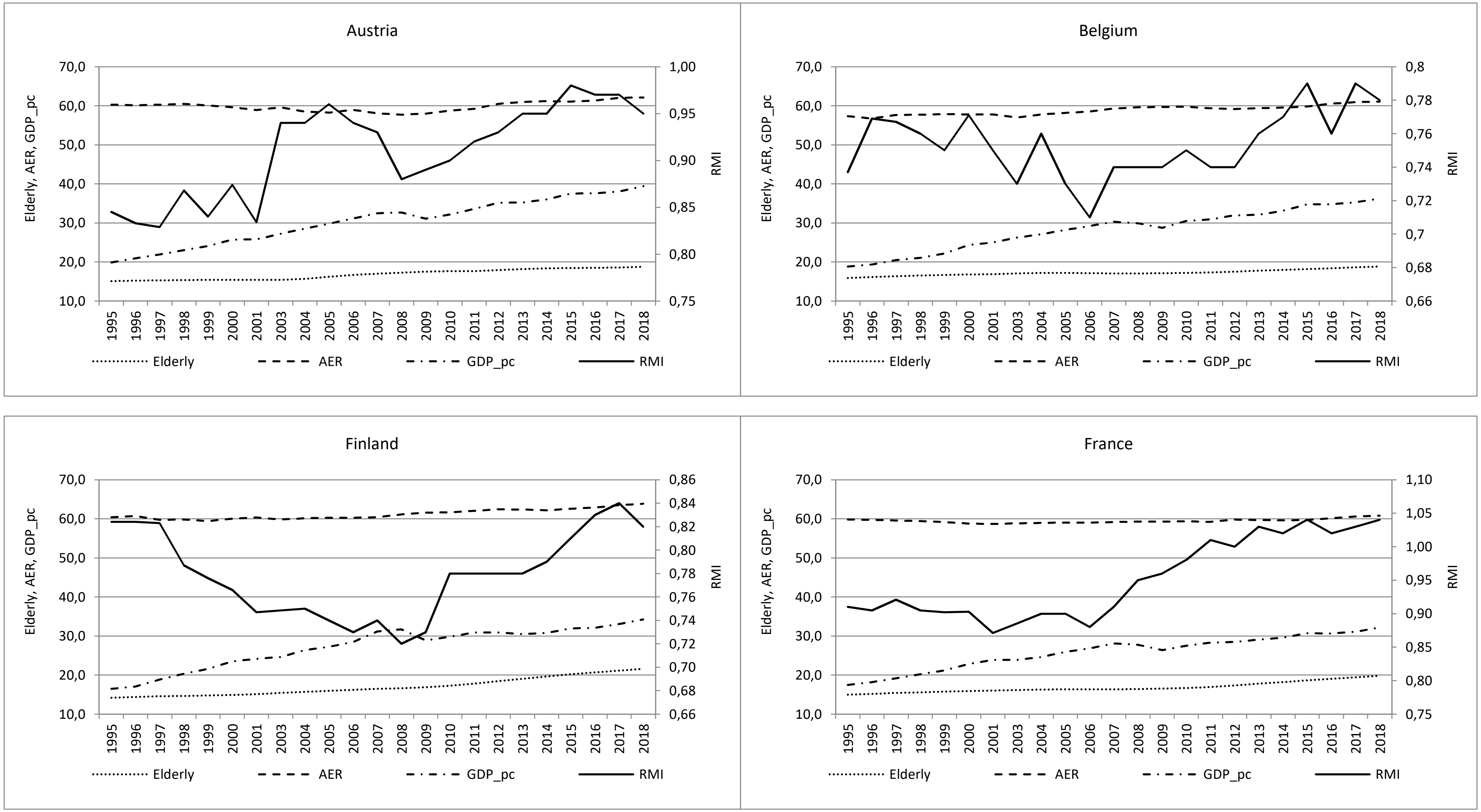

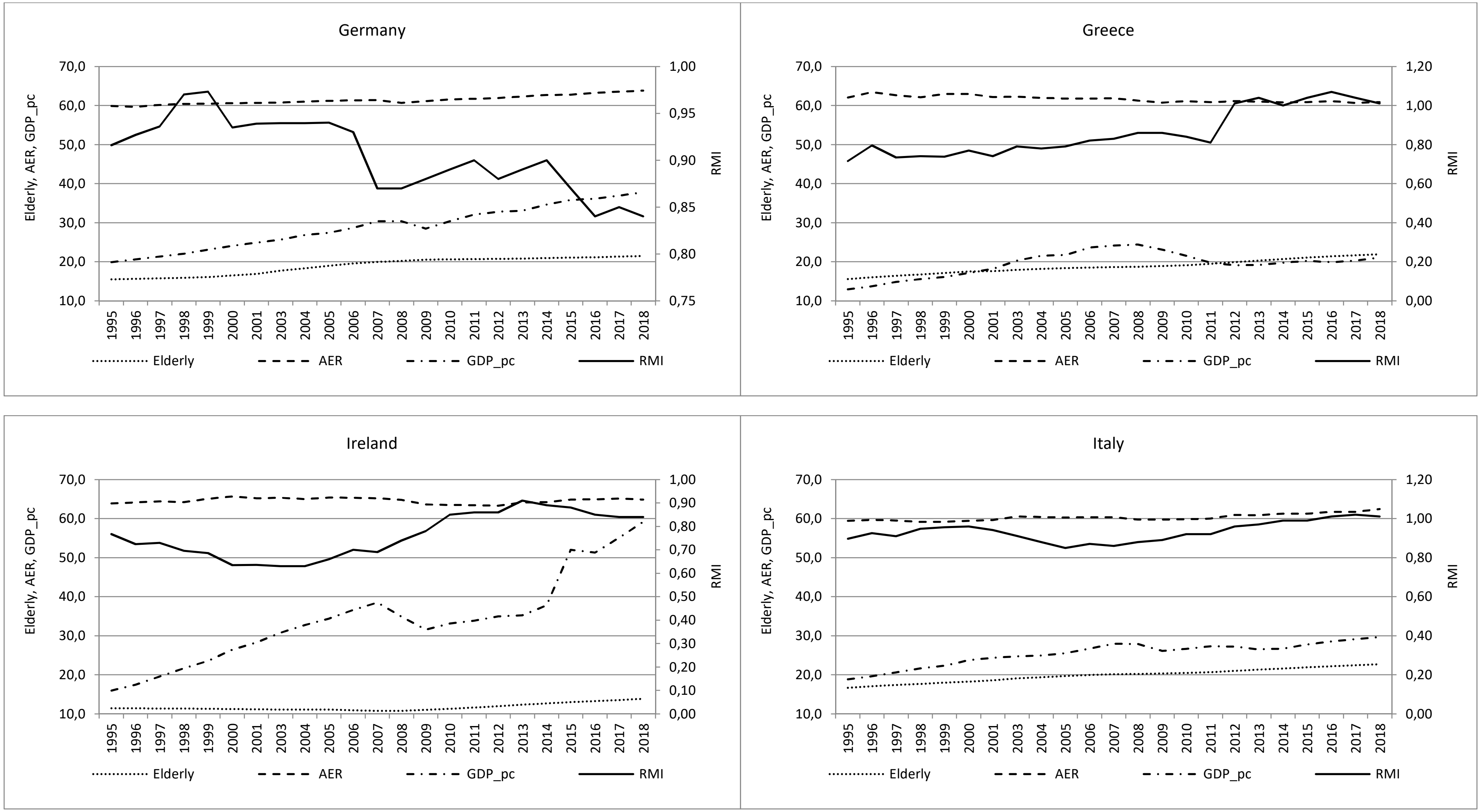
Germany
Greece
Ireland
Italy
Elderly, AER, GDP_pc
RMI
70,0
60,0
50,0
40,0
30,0
20,0
10,0
1,20
1,00
0,95
0,90
0,85
0,80
0,75
0,60
0,40
0,20
0,00
1995 1996 1997 1998 1999 2000 2001 2003 2004 2005 2006 2007 2008 2009 2010 2011 2012 2013 2014 2015 2016 2017 2018
Elderly
AER
GDP_pc
RMI

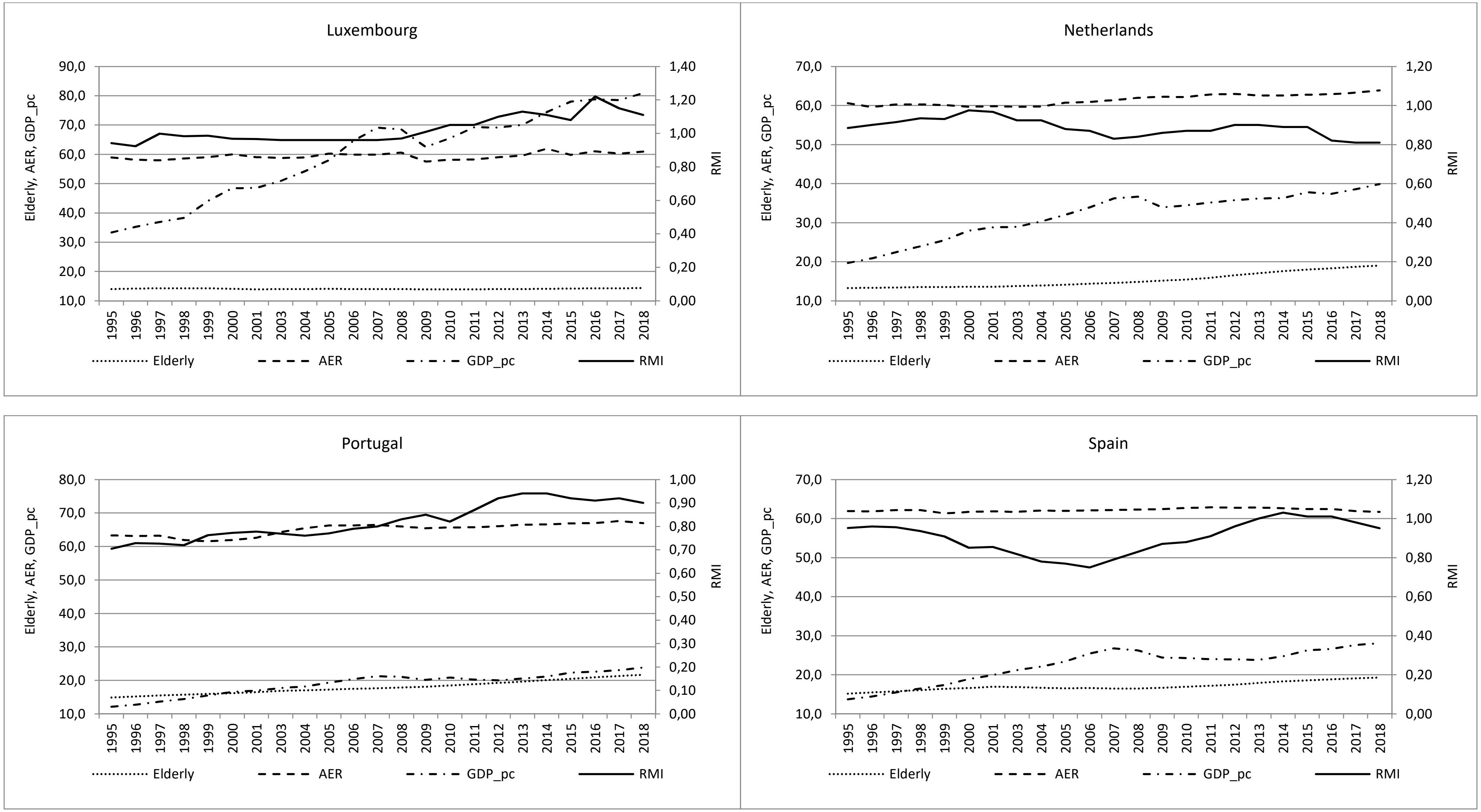
Luxembourg
90,0
80,0
70,0
60,0
50,0
40,0
30,0
20,0
10,0
1,40
1,20
1,00
0,80
0,60
0,40
0,20
0,00
Elderly, AER, GDP_pc
RMI
1995 1996 1997 1998 1999 2000 2001 2003 2004 2005 2006 2007 2008 2009 2010 2011 2012 2013 2014 2015 2016 2017 2018
Elderly
AER
GDP_pc
RMI
Netherlands
70,0
60,0
50,0
40,0
30,0
20,0
10,0
1,20
1,00
0,80
0,60
0,40
0,20
0,00
Elderly, AER, GDP_pc
RMI
1995 1996 1997 1998 1999 2000 2001 2003 2004 2005 2006 2007 2008 2009 2010 2011 2012 2013 2014 2015 2016 2017 2018
Elderly
AER
GDP_pc
RMI
Portugal
80,0
70,0
60,0
50,0
40,0
30,0
20,0
10,0
1,00
0,90
0,80
0,70
0,60
0,50
0,40
0,30
0,20
0,10
0,00
Elderly, AER, GDP_pc
RMI
1995 1996 1997 1998 1999 2000 2001 2003 2004 2005 2006 2007 2008 2009 2010 2011 2012 2013 2014 2015 2016 2017 2018
Elderly
AER
GDP_pc
RMI
Spain
70,0
60,0
50,0
40,0
30,0
20,0
10,0
1,20
1,00
0,80
0,60
0,40
0,20
0,00
Elderly, AER, GDP_pc
RMI
1995 1996 1997 1998 1999 2000 2001 2003 2004 2005 2006 2007 2008 2009 2010 2011 2012 2013 2014 2015 2016 2017 2018
Elderly
AER
GDP_pc
RMI

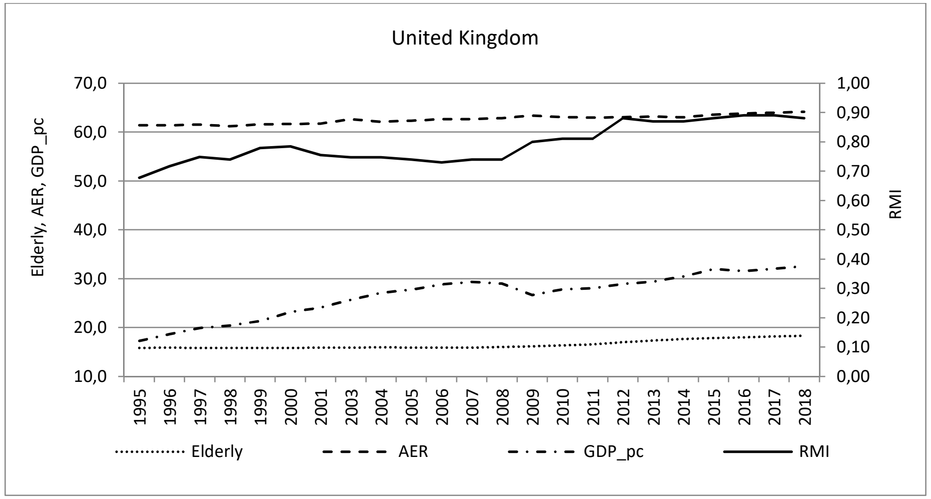
United Kingdom
70,0
60,0
50,0
40,0
30,0
20,0
10,0
Elderly, AER, GDP_pc
1,00
0,90
0,80
0,70
0,60
0,50
0,40
0,30
0,20
0,10
0,00
RMI
1995
1996
1997
1998
1999
2000
2001
2003
2004
2005
2006
2007
2008
2009
2010
2011
2012
2013
2014
2015
2016
2017
2018
Elderly
AER
GDP_pc
RMI

Table A1. The results of Chow test for structural break

Country	F(2,19) statistics	*p*-value
Mean (for all the countries)	24.2769	<0.01
Austria	8.8779	<0.01
Belgium	10.8722	<0.01
Finland	61.7709	<0.01
France	41.2185	<0.01
Germany	1.1949	0.3245
Greece	5.0723	0.0172
Ireland	17.2034	<0.01
Italy	26.5311	<0.01
Luxembourg	10.3205	<0.01
Netherlands	0.0018	0.9982
Portugal	2.9569	0.0762
Spain	48.2187	<0.01
United Kingdom	6.972	<0.01

Source: own computations on the basis of OECD and Eurostat data
